# Related factors of periodontal health among Chinese middle school students, findings from a national cross-sectional survey

**DOI:** 10.1186/s12903-021-01889-2

**Published:** 2021-10-12

**Authors:** Jingyu Zhan, Yu Zhang, Xing Wang, Baojun Tai, Deyu Hu, Huancai Lin, Bo Wang, Yan Si, Chunxiao Wang, Shuguo Zheng, Xuenan Liu, Wensheng Rong, Weijian Wang, Xiping Feng, Xi Chen

**Affiliations:** 1 Shanghai Key Laboratory of Stomatology and Shanghai Research Institute of Stomatology, Department of Preventive Dentistry, Shanghai Ninth People’s Hospital, College of Stomatology, National Clinical Research Center for Oral Diseases, Shanghai Jiao Tong University School of Medicine, 639 Zhizaoju Road, Shanghai, People’s Republic of China; 2 Chinese Stomatological Association, Beijing, People’s Republic of China; 3 School and Hospital of Stomatology, Wuhan University, Wuhan, People’s Republic of China; 4 West China Hospital of Stomatology, Sichuan University, Chengdu, People’s Republic of China; 5 Guanghua School of Stomatology, Hospital of Stomatology, Sun Yat- Sen University, Guangzhou, People’s Republic of China; 6 Peking University School and Hospital of Stomatology, Beijing, People’s Republic of China; 7 Chinese Center for Disease Control and Prevention, Beijing, People’s Republic of China

**Keywords:** Gingival bleeding, Calculus, Adolescent, Generalized linear mixed model

## Abstract

**Background:**

To investigate the related risk factors of periodontal health status among Chinese middle school students.

**Methods:**

This study is a part of the Fourth National Oral Health Epidemiological Survey, which is by far the largest oral epidemiological survey in China, including all provinces, municipalities and autonomous regions in mainland China. A multi-stage stratified sampling method was used to select middle school students aged 12–15 from the sampled middle school for investigation. The survey consisted of two parts: oral examination and questionnaire survey. The oral examination included gingival bleeding and calculus. The questionnaire included sociodemographic information, oral health knowledge, attitudes and behaviors. Logistic regression and generalized linear mixed model were used to investigate the risk factors of gingival bleeding and calculus.

**Results:**

A total of 118,514 middle-school students has been examined. Less gingival bleeding (OR = 0.746, CI 0.718–0.774) and calculus (OR = 0.550, CI 0.529–0.527) were found in 12-year-old group compared to 15-year-old group. The periodontal health status of males was worse than that of females (gingival bleeding OR = 1.102, CI 1.074–1.132, calculus OR = 1.258, CI 1.223–1.295). Besides age and gender, region, living place, ethnic groups, family structure, parent’s education level, oral health knowledge and behavior were also related to gingival bleeding and calculus.

**Conclusions:**

Gingival bleeding and calculus occurred most of 12–15 years old adolescents in China. Several related factors, such as gender, age, ethnicity, father’s education level, oral health knowledge and behavior, were found in multi-factorial models. The impact of province should arouse people’s attention.

## Background

Severe periodontitis, affecting approximately more than one-tenth of whole population in the world, is one of the most common oral diseases [1]. It is regarded as a public health problem due to the high prevalence, impairing the quality of life and causing disability [2, 3]. Periodontitis represents a range of clinical manifestations from mild subclinical inflammation to advanced destructive forms, leading to tooth loss [4]. Diagnosis is based mainly on clinical assessment of surrogate markers such as probing pocket depth (PD) and clinical attachment level (CAL) and radiographic evidence of alveolar bone loss. Gingival bleeding and calculus are the prelude of occurrence of periodontal disease and they are also common in adolescents [5].

For the middle-school students, the periodontal problems usually performed as plaque, calculus, gingival bleeding, while shallow pocket depth and attachment loss were not common [6]. On the other hand, these periodontal diseases maintained at an early stage and reversible. In the group of 15–16-year-old adolescents of Finland, the prevalence of early periodontitis was between 10 and 15% [7].

The national oral health survey was conducted every 10 years and covered all provinces, municipalities, and autonomous regions. In the latest survey, it was found that gingival bleeding and calculus occurred more than six out of ten adolescents. In the 15-year-old population, 6.5% adolescents had periodontal pocket and 0.5% had attachment loss [8].

To investigate and further analyze the risk factors of Chinese adolescent’s periodontal health, multi-factorial models of gingival bleeding and calculus were established in this study.

## Methods

This study protocol was approved by the Ethics Committee of the Chinese Stomatological Association (Approval no. 2014-003). It was conducted from August 2015 to December 2016 in all 31 provinces, autonomous regions and municipalities of mainland China. Informed consent was obtained from all subjects or, if subjects are under 18, from a parent and/or legal guardian. All methods were carried out in accordance with relevant guidelines and regulations.

### Sampling

In this study, sample size was calculated based on the data of third national oral health survey, in which the prevalence of gingival bleeding, calculus and dental caries were 59.1%, 57.7% and 28.9%, respectively. Since this is a comprehensive oral health survey not only concerning periodontal disease, the lowest number of dental caries was chosen. The 95% confidence interval was set to 15% in 12–15 years old group and the designed effect was set to 4.5. To meet the response rate of 80%, the final sample size was 28,365 each for the 12–15 years age groups.

A multistage cluster sampling was used in this study. First of all, two districts and two counties in each province were chosen randomly according to probability proportional to size (PPS) sampling. Secondly, three middles schools were selected in each district or county. Thirdly, 80 students in every age group were asked to participate in this survey by cluster sampling method.

### Clinical examination and questionnaire survey

Gingival bleeding and calculus status were scored as 0 or 1 corresponding to absence or presence, respectively following the method of modified community periodontal index (CPI) recommended by WHO. Calibration training was carried out to ensure the reliability before the field work. Every clinical examiner was calibrated with a standard examiner and the kappa value was required to be higher than 0.6. At the survey site, 5% of subjects were randomly selected to finish the duplication for the surveillance of inter-examiner reliability. The kappa value was also required to be higher than 0.6.

A self-completed structured questionnaire was applied to collect data of participants’ sociodemographic information (gender, age, etc.), oral health attitude, knowledge and behaviors (daily use of tooth brush and dental floss). Teachers and researchers would explain the content of this questionnaire before the adolescents completed the questions and answered the questions when they filled in the questionnaire.

### Data analysis

All collected data was input into a dedicated National Survey data management system, and data of each subject was fed into computer for two times. If the two entries were inconsistent, the system would automatically give a reminder and the entry personnel would check and confirm correct data immediately. During the data entry process, logical check and normal value ranges were strictly preset.

Bivariate analysis was performed to investigate the relationship between periodontal status and relevant variables. The dependent dichotomous variables were gingival bleeding and calculus scored 0 or 1. The independent variables were age, gender, region (east, middle and west), resident location (urban or rural), nation, family structure (one-child family or not), father and mother’s educational level, smoking habit, brush teeth, using dental floss, oral health attitude and knowledge. All independent variables which were significant in the chi-square test were selected in the original binary logistic regression model (backward stepwise). Odds ratio with 95% confidence interval was calculated to investigate the association among the periodontal status with other potential risk factors. As the random effect of the province variable, generalized linear mixed model (GLMM) was introduced in this study. The variable province was regarded as a fixed variable and the other significant relevant variables were entered in GLMM model. All the statistical significance was set as *P* < 0.05.

## Results

Totally 118,514 subjects (14.0 ± 1.09 years old) participated in this survey. The prevalence of gingival bleeding and calculus was 61.0% and 67.3% respectively. With the increasing of the age, the periodontal status became worse (*P* < 0.001).

In the binary analysis, there were 13 variables associated with periodontal health, which were included in the logistic regression to analyze the multi-factorial effect on gingival bleeding. In the final model (Table 1), it was found that gender, age, region, living place, nation, family structure, parents’ education level, dental hygiene habits and oral health knowledge were associated with gingival bleeding. The results were consistent with the binary analysis.

**Table 1:** Univariate and stepwise logistic regression on possible risk factor for gingival bleeding

Factor	Univariate					Stepwise		
	*p* value	OR	95% CI for OR		*p* value	OR	95% CI for OR	
Gender								
Male	< 0.001	1.12	1.09	1.15	< 0.001	1.11	1.08	1.14
Female^a^								
Age	< 0.001				< 0.001			
12		0.77	0.74	0.79	< 0.001	0.75	0.72	0.78
13		0.82	0.79	0.85	< 0.001	0.81	0.78	0.84
14		0.85	0.82	0.88	< 0.001	0.83	0.80	0.86
15^a^								
Region	< 0.001				< 0.001			
East		0.81	0.79	0.84	< 0.001	0.86	0.83	0.89
West		1.19	1.15	1.22	.004	1.05	1.02	1.09
Central^a^								
Living place								
Urban	< 0.001	1.06	1.04	1.09	0.001	1.10	1.07	1.13
Rural^a^								
Ethnic group								
Han	< 0.001	0.67	0.65	0.70	< 0.001	0.77	0.74	0.80
Minority^a^								
Family structure								
One-child family	< 0.001	0.74	0.73	0.76	< 0.001	0.81	0.79	0.84
With siblings^a^								
Father education	< 0.001				.001			
Middle school		1.50	1.44	1.56	.005	1.09	1.03	1.15
High school		1.29	1.25	1.34	< 0.001	1.09	1.04	1.15
Univeristy^a^								
Mother education	< 0.001				.045			
Middle school		1.50	1.44	1.56	.041	1.06	1.00	1.12
High school		1.25	1.21	1.30	.397	1.02	.97	1.07
Univerisity^a^								
Smoking								
Yes	< 0.001	1.20	1.15	1.26				
No^a^								
Brush teeth								
Yes	< 0.001	0.64	0.61	0.66	< 0.001	0.69	0.66	0.73
No^a^								
Dental floss								
Yes	< 0.001	0.81	0.78	0.84	< 0.001	0.89	0.85	0.93
No^a^								
Oral health knowledge	< 0.001				< 0.001			
0–3		1.32	1.28	1.36	< 0.001	1.21	1.16	1.25
4–5		1.18	1.15	1.22	< 0.001	1.12	1.09	1.16
6–8^a^								
Oral health attitude								
Negative	0.002	1.04	1.02	1.97				
Positive^a^								
Constant					< 0.001	2.83		

^a^Reference category

Males have more chance of gingival bleeding and the elder adolescents are more likely to have gingival bleeding than younger subjects. Adolescents living in eastern China relatively are not as susceptible as ones in western China. Subjects from one-child family and Han population have less chance of gingival bleeding. Fewer adolescents with higher educational parents suffered gingival bleeding. Good oral hygiene habit (brushing tooth) displayed greatest effect on prevent gingival bleeding. The adolescents who got lower oral health knowledge scores had higher chance of gingival bleeding.

Very similar results were shown in the Table 1, which presented the logistic regression model of calculus and associated factors (Table 2). With the increasing of age, more calculus was found in the mouth. The differences among regions became greater. And besides the same risk factors compared to the model of gingival bleeding, smoking habit was added. The smokers were more likely to have calculus than non-smokers.

**Table 2:** Univariate and stepwise logistic regression on possible risk factor for calculus

Factor	Univariate					Stepwise		
	*p* value	OR	95% CI for OR		*p* value	OR	95% CI for OR	
Gender								
Male	< 0.001	1.26	1.23	1.29	< 0.001	1.26	1.22	1.30
Female^a^								
Age	< 0.001				< 0.001			
12		0.57	0.55	0.59	< 0.001	0.55	0.53	0.57
13		0.66	0.64	0.68	< 0.001	0.64	0.62	0.67
14		0.81	0.79	0.84	< 0.001	0.80	0.77	0.83
15^a^								
Region	< 0.001				< 0.001			
East		0.74	0.72	0.76	< 0.001	0.82	0.79	0.85
West		1.50	1.45	1.54	< 0.001	1.39	1.34	1.45
Central^a^								
Living place								
Urban	0.001	0.96	0.94	0.98	.069	1.02	1.00	1.06
Rural^a^								
Ethnic group								
Han	< 0.001	0.66	0.64	0.69	.001	0.92	0.88	0.97
Minority^a^								
Family structure								
One-child family	< 0.001	0.68	0.66	0.69	< 0.001	0.78	0.76	0.81
With siblings^a^								
Father education	< 0.001				.002			
Middle school		1.86	1.78	1.94	.001	1.11	1.04	1.17
High school		1.40	1.35	1.45	.085	1.04	0.99	1.10
University^a^								
Mother education	< 0.001				< 0.001			
Middle school		1.90	1.82	1.98	< 0.001	1.21	1.14	1.28
High school		1.39	1.34	1.44	.001	1.09	1.04	1.15
University^a^								
Brush teeth								
Yes	< 0.001	0.61	0.58	0.63	< 0.001	0.69	0.66	0.73
No^a^								
Dental floss								
Yes	< 0.001	0.67	0.64	0.69	< 0.001	0.78	0.75	0.82
No^a^								
Smoking								
Yes	< 0.001	1.59	1.51	1.68	.009	1.08	1.02	1.15
No^a^								
Oral health knowledge					< 0.001			
0–3		1.25	1.21	1.29	< 0.001	1.10	1.06	1.14
4–5		1.24	1.20	1.27	< 0.001	1.13	1.09	1.16
6–8^a^								
Oral health attitude								
Negative	0.001	1.04	1.02	1.07				
Positive^a^								
Constant					< 0.001	3.30		

^a^Reference category

The oral health knowledge and attitude of adolescents varies among provinces. For example, in some provinces, the proportion of adolescents with high scores in oral health knowledge (≥ 6 right answers of 8 questions) was less than 20%, but in the highest provinces it exceeded 60%. The highest and lowest proportion of adolescents with positive oral health attitude was 73.9% and 49.3% respectively grouped by province. GLMM was applied to minimize the influence of province.

The results of GLMM showed that gender, age, living place, nation, father’s education level, smoking, oral health knowledge and behavior were associated with gingival bleeding (Table 3). Male adolescents were more likely to have gingival bleeding. With the increasing of age, the risk of gingival bleeding increased. The subjects who lived in urban area were more likely to have gingival bleeding. Adolescents from Han ethnic group were less susceptible to gingival bleeding compared to those from other ethnic groups. Subjects with better oral hygiene behaviors were less likely to have bleeding on gingival. Gingival bleeding was less likely to occur among the subjects who got higher score in oral health knowledge.

**Table 3:** The generalized linear mixed model of gingival bleeding

	Coefficient	Sig.	Exp (coefficient)	95% CI for exp (coefficient)	
				Lower	Upper
Intercept	0.87		2.39	1.52	3.75
Gender					
Male	0.11	< 0.001	1.12		
Female^a^				1.09	1.15
Age					
12	− 0.35	< 0.001	0.70	0.67	0.73
13	− 0.26	< 0.001	0.77	0.74	0.80
14	-0.22	< 0.001	0.80		
15^a^				0.77	0.83
Living place					
Urban	0.18	< 0.001	1.20		
Rural^a^				1.16	1.23
Ethnic group					
Han	− 0.08	.003	0.92	0.87	0.97
Minority					
Father education					
Middle school	0.18	< 0.001	1.20	1.140	1.26
High school	0.12	< 0.001	1.14	1.09	1.18
University^a^					
Brush teeth					
Yes	− 0.29	< 0.001	0.75	0.71	0.79
No^a^					
Dental floss					
Yes	− 0.20	< 0.001	0.82	0.78	0.86
No^a^					
Oral health knowledge					
0–3	0.09	.002	1.09	1.05	1.13
4–5	0.06	.001	1.06		
6–8^a^				1.03	1.10

^a^Reference category

The model of calculus was shown in Table 4. There were eight related factors and seven were same as those in gingival bleeding model. The same variables were gender, age, ethnic group, father education level, brush teeth, dental floss and oral health knowledge. The factor living place was not in this model compared with the model of gingival bleeding, and family structure seemed to present good effect on minimizing calculus. Participant’s oral health attitude was not included in both generalized linear mixed model of gingival bleeding and calculus.

**Table 4:** The generalized linear mixed model of calculus

	Coefficient	Sig.	Exp (coefficient)	95% CI for exp (coefficient)	
				Lower	Upper
Intercept	1.17		3.23	2.34	4.47
Gender					
Male	0.28	< 0.001	1.32	1.28	1.36
Female^a^					
Age					
12	− 0.67	< 0.001	0.51	0.49	0.53
13	− 0.48	< 0.001	0.62	0.59	0.64
14	− 0.25	< 0.001	0.78	0.75	0.82
15^a^					
Ethnic group					
Han	− 0.10	.003	0.91	0.86	0.96
Minority^a^					
Family structure					
One-child family	− 0.13	< 0.001	0.88	0.85	0.91
With siblings^a^					
Father education					
Middle school	0.23	< 0.001	1.26	1.20	1.33
High school	0.11	< 0.001	1.12	1.08	1.17
University^a^					
Brush teeth					
Yes	− 0.11	< 0.001	0.90	0.85	0.95
No^a^					
Dental floss					
Yes	− 0.18	< 0.001	0.83	0.79	0.87
No^a^					
Oral health knowledge					
0–3	0.06	.002	1.06	1.02	1.11
4–5	0.06	.001	1.06	1.03	1.09
6–8^a^					

^a^Reference category

## Discussion

The periodontal condition of Chinese adolescents was relatively unsatisfying, which was not improved compared to the situation 10 years ago. Most of the survey respondents had problems with gingival bleeding and calculus, which reflected that both parents and adolescents themselves lacked sufficient attention to periodontal health. The results of multivariate analysis showed that age, region, place of residence, family structure, parents ‘education level, oral health behavior, and respondents’ oral health knowledge were related to gingival bleeding and calculus. After adjusting the province’s influence on the results, factors such as gender, age, ethnicity, father’s education level, oral health knowledge and behavior were related to adolescent periodontal status.

The differences of periodontal status among different gender and age groups were found in several studies and consistent with the results in past national surveys [9, 10]. Oral health behavior and knowledge also direct affected personal periodontal health [11, 12]. The socio-economic factors, e.g. region, ethnical group, parents’ education level, were related to adolescent’s gingival bleeding and calculus.

The large difference among eastern, middle and western regions still existed. The periodontal status of teenagers in eastern region was obviously better than those in western region. There is a huge imbalance of economic development and medical resources among different regions in China. Periodontal diseases were more common in the less developed regions [13]. As the residences in the most developed region in China, adolescents in eastern region had greatly lower risk of periodontal disease compared to those in middle and western regions. A gratifying decline in the occurrence of gingival bleeding and calculus in the middle region has been achieved in the past decade [9]. This might be related to its quick development on economics and enhancement of oral health resources in twenty-first century.

Han nation is the major nation with the largest population in China and the other 55 minority nations constitute about 10% of the population, around 140 million. In the previous surveys regarding the periodontal status of ethnic minorities in China, it was found that 93% of Dai children in 12 years old had gingivitis and 71% of Bulang children had bleeding gum [14]. The adolescents of ethnic minorities in China had a higher risk of periodontal disease compared to those of Han nation. Most of the ethnic minorities stay in rural area of middle and western regions, where dental resource is insufficient. The comparatively lower socio-economical level aggravates the inequality on dental resource [15].

China is a vast and densely populated country, where the differences in economic development level among different provinces and cities were huge. For example, cities with high levels of economic development have a per capita GDP of more than US $ 20,000, which is close to that of developed countries. However, the province with the worst economic development is only a quarter of the formers, and it is still underdeveloped [10]. In addition, many provinces and cities have different living habits, education levels and medical resources. Therefore, it was relatively simple and rough to divide all provinces, autonomous regions, and municipalities into three regions (east, middle, and west) according to the geographical location and the degree of economic development. In order to get further understand whether the differences among provinces and cities affect the periodontal status of adolescents, we also used the province as a separate variable to sort out the relevant influencing factors of adolescents’ periodontal status. The generalized linear mixed model was applied while province was considered as a random variable [16].

After the adjustment of province, several common factors left in gingival bleeding and calculus models (shown in Table 3, 4). This result gave us a great hint that, in China, the impact of different provinces might exceed the impact of individual factors. The province variable is a variable integrates multiple economic factors, such as ethnicity, family structure, and other factors. Therefore, it was not only necessary to explore common influences across the country, various situations in different provinces should also be taken into consideration.

This study had several limitations. First of all, this was a cross-sectional study, which could not identify the cause-effect relationship between risk factors and outcomes. However, the risk factors found in this study would provide information for the further longitudinal study. Second, CPI was used in this study. Community Periodontal Index (CPI) was originated from Community Periodontal Index of Treatment Needs (CPITN) and modified to include all teeth in all four quadrants [17]. CPI/CPITN was not sensitive for the prevalence estimate of PD ≥ 6 mm and also not accurate enough for the estimates of periodontitis extent [18]. This index was design for large-scale screening but not diagnosed for gingivitis and periodontitis [19]. Thus, it was hard to estimate the prevalence of gingivitis and periodontitis of Chinese adolescents from this study.

Gingival bleeding is the primary symptom of gingival disease. The high prevalence of gingival bleeding and calculus indicated the potential risk of gingivitis and poor oral hygiene in Chinese adolescents. If this situation did not attract public’s attention, more patients with periodontal disease would appear in the future. China has been facing severe periodontal disease and tooth loss status in adult and elderly populations. The prevalence of clinical attachment loss (CAL > 3 mm) was 33.2% among 35–44-year-old Chinese adults [20], and mean of remaining teeth was 22.5 in 65–74-year-old group [21]. Effort and support on oral health shall been delivered to adolescents to avoid same similar situation happening again in next generation.

## Conclusions

Gingival bleeding and calculus occurred most of 12–15 years old adolescents in China. Several related factors, such as gender, age, ethnicity, father’s education level, oral health knowledge and behavior, were found in multi-factorial models. The impact of province should arouse people’s attention.

## Data Availability

All data generated or analyzed during this study are included in this published article.

## Abbreviations

PD: Probing pocket depth
CAL: Clinical attachment level
CPI: Community periodontal index
